# Renal resistive index and long-term outcome in patients with coronary artery disease

**DOI:** 10.1186/s12872-020-01607-w

**Published:** 2020-07-06

**Authors:** Maciej T. Wybraniec, Maria Bożentowicz-Wikarek, Magdalena Olszanecka-Glinianowicz, Jerzy Chudek, Katarzyna Mizia-Stec

**Affiliations:** 1 First Department of Cardiology, School of Medicine in Katowice, Medical University of Silesia, 47 Ziołowa St., 40-635 Katowice, Poland; 2 Department of Pathophysiology, School of Medicine in Katowice, Medical University of Silesia, Katowice, Poland; 3 Department of Internal Medicine and Oncological Chemotherapy, Medical University of Silesia, Katowice, Poland

**Keywords:** Coronary artery disease, Renal resistive index, Renal pulsatility index, Major adverse cardiovascular events coronary artery disease

## Abstract

**Background:**

The study aimed to evaluate the application of intra-renal Doppler flow indices for the prediction of major adverse cardiac and cerebrovascular events (MACCE) during 24-month follow-up in patients with coronary artery disease (CAD) subject to coronary angiography (CA).

**Methods:**

This prospective study comprised 111 consecutive patients with stable and unstable CAD (68.5% men; median age 65 years), referred for CA. Ultrasonographic parameters of intra-renal blood flow in arcuate/interlobular arteries, including renal resistive index (RRI) and pulsatility index (RPI), were acquired directly before and 1 h after the procedure. Endpoint of MACCE (cardiovascular death, myocardial infarction, myocardial revascularization or stroke) were recorded during 24-month follow-up.

**Results:**

MACCE occurred in 14 patients (12.6%). Patients with MACCE had more diffuse CAD reflected by Syntax score (23.6 vs.14.4 pts., *p* = 0.02), higher platelet level (242.4 vs. 207.2 × 1000/μl, *p* = 0.01), higher rate of left main CAD (42.9% vs.5.2%, *p* < 0.001) and left ventricular ejection fraction < 50% (50% vs.23.7%,*p* = 0.045). Patients with MACCE had higher pre-procedural (0.68 ± 0.06 vs. 0.62 ± 0.06, *p* < 0.001) and post-procedural RRI (0.72 ± 0.06 vs.0.66 ± 0.06, *p* = 0.01), but comparable RPI (*p* = 0.63 and *p* = 0.36, respectively). Cox proportional hazards model revealed that pre-procedural RRI (OR = 1.11 per 0.01; *p* = 0.02) and left main CAD (OR = 5.75, *p* = 0.002) were the only independent predictors of MACCE occurrence. Receiver operator characteristic curve analysis revealed that preprocedural RRI > 0.645 accurately predicted the composite endpoint (AUC = 0.78, *p* = 0.001) and identified patients with impaired 24-month prognosis according to Kaplan-Meier curve (log-rank *p* < 0.001).

**Conclusions:**

Increased pre-procedural RRI, together with left main CAD, are associated with worse 24-month prognosis in patients with CAD referred for CA.

## Background

In contrast to ST-segment elevation myocardial infarction, chronic coronary syndromes (CCS) and non-ST-segment elevation acute coronary syndromes (NSTE-ACS) share different set of predictors of long-term outcome [1]. Numerous well-established clinical prognostic factors in NSTE-ACS patients exists, such as age, left ventricular ejection fraction (LVEF), completeness of revascularization, SYNTAX score [2], presence of acute heart failure, peak troponin elevation and ST-segment deviation [3]. Still, baseline renal function represents a central determinant of long-term mortality, reflecting the vital interplay between heart and kidneys [4]. The coexistence of cardiac and renal pathology, known as cardio-renal syndrome [5], is linked to humoral and neural signaling, and extends far beyond mere coincidence. The acute and chronic cardio-renal syndrome is partially modulated via catecholamine surge and increased sympathetic tone [5]. Although sympathetic nervous system hyperactivity has been deemed responsible for impaired survival [6], its evaluation is cumbersome and not amenable to routine clinical practice [7].

Renal vascular hemodynamics provides an indirect measure of sympathetic nervous function and can be easily assessed by means of Doppler ultrasound analysis of the spectrum of flow in interlobular and/or arcuate arteries [8]. Among numerous parameters, renal resistive index (RRI) was shown to be the most reproducible and clinically meaningful indices of both renal vascular resistance and stiffness [9, 10]. RRI was shown to be dependent on numerous factors, including renal artery stiffness, age, pulse blood pressure, severe brady- and tachycardia, presence of valvular heart disease or any pathological lesions within renal parenchyma [11]. In daily clinical practice RRI may facilitate the diagnosis of renal artery stenosis [12] or help diagnose an acute rejection in kidney transplant recipients [13]. Assessment of pre-procedural RRI allowed for early identification of patients at risk of contrast-induced acute kidney injury (CI-AKI) among patients with coronary artery disease (CAD) referred for coronary angiography (CA) [14]. RRI also accurately predicted the onset [15] and persistence of acute kidney injury [16]. Moreover, RRI appears to be a general measure of arterial remodeling and stiffness and was linked to central pulse pressure values and echocardiographic parameters of left ventricular systolic and diastolic blood flow [10]. Accordingly, parameters of intra-renal blood flow were independently associated with occurrence of cardiovascular adverse events in an unselected elderly population [17] and RRI specifically, among patients with essential hypertension, especially in those with hypertensive renal disease [18]. Of note, in a large cohort of patients with chronic kidney disease (CKD), elevated RRI was unequivocally associated with increased mortality [19]. Yet, none of the previously published studies evaluated RRI as a prognostic factor in patients with CAD undergoing cardiac catheterization.

The study aimed to verify the hypothesis that the use of intra-renal flow parameters may identify patients with coronary artery disease (CAD) at risk of major adverse cardiac and cerebrovascular events (MACCE) during 24-month follow-up.

## Methods

The study represents a prospective observation of different predictors of adverse cardiovascular events among patients with CAD referred to elective or urgent coronary angiography (CA), with special consideration of intra-renal Doppler flow parameters. The study covered consecutive one hundred and eleven patients with the clinical suspicion of CAD who met inclusion criteria of either: a) stable coronary syndrome with clinical indication for CA based on former non-invasive stress test or b) non-ST-segment elevation acute coronary syndrome (NSTE-ACS). The enrollment phase took place between 2014 and 2016.

The exclusion criteria involved cardiogenic shock, pulmonary edema, any sort of respiratory failure, chronic kidney disease (eGFR < 50 ml/min/1.73 m^2^ or proteinuria > 500 mg/l), renal structural abnormalities, active urinary tract infection, renal artery stenosis, pregnancy, previous hypersensitivity to contrast medium, morbid obesity (body mass index, BMI > 40 kg/m^2^). In addition, exclusion criteria entailed conditions that could alter the intra-renal hemodynamics, such as moderate to severe aortic valve stenosis, severe valvular heart disease of any kind, high pulse pressure > 80 mmHg, tachycardia > 100 bpm or bradycardia < 50 bpm.

The study was carried out in adherence to the principles of the Declaration of Helsinki and was formerly assessed and approved by local Ethics Committee. All study participants gave written informed consent to study participation.

The study protocol involved meticulous review of demographic and clinical data, as well as acquisition of blood specimen for rudimentary laboratory tests at baseline, including serum creatinine concentration (SCr). Furthermore, 24-h and 48-h blood samples were assayed for SCr in order to facilitate possible diagnosis of CI-AKI.

All the patients underwent renal ultrasound study with Doppler analysis of indices of blood flow within arcuate or interlobular arteries. The study was performed twice: directly before and 1 h following coronary angiography. The evaluation was conducted by a single experienced investigator by means of Vivid 7 (GE Healthcare) with a 5C probe (4.4–6.7 MHz). Prior to the measurements, patients were required to remain supine for at least 10 min. First, the morphology of kidneys was assess in order to establish their length and width, as well as potential structural abnormalities precluding further evaluation. Subsequently, patients were screened for renal artery stenosis using peak systolic and end-diastolic flow velocity within the main renal artery. A 2–4 mm pulse-wave Doppler gate was applied for intra-renal arteries in order to assess a set of indices, including peak systolic (PSV) and end-diastolic velocity (EDV), mean velocity (MV), acceleration time (AT), augmentation index (AI). The above-mentioned parameters were then used to calculate renal resistive index (RRI) and renal pulsatility index (RPI) based on well-known formulas: RRI = (PSV–EDV)/PSV and RPI = (PSV–EDV)/MV. The core indices were acquired 3 times on each side during both pre- and post-procedural evaluation. The final pre- and post-procedural values constituted arithmetic means of these measurements.

### Follow-up and primary endpoint

Patients were followed up for 24-months using a structured telephone interview performed 1-month, 12-months and 24-months after the hospitalization. Furthermore, data on any available outpatient visit or recurrent hospital admission in the local electronic database was gathered.

The primary endpoint comprised all major adverse cerebral and cardiovascular events (MACCE), including cardiovascular death, myocardial infarction, need for urgent revascularization and ischemic stroke.

### Peri-procedural management

All study participants were treated in line with the European Society of Cardiology 2014 Guidelines on Myocardial Revascularization [20]. The coronary angiography was performed using low- or iso-osmolar contrast agents. Although patients with CKD defined as eGFR < 50 ml/min/1.73 m^2^ were excluded from the study, patients with borderline renal function (eGFR 50–60 ml/min) obtained an intravenous drip of 0.9% saline (1 ml/kg/h; 12 h before - 24 after coronary angiography). Patients with preserved kidney function (eGFR > 60 ml/min) received one 500 ml 0.9% saline before the procedure. Biguanides were stopped 24 h prior to contrast.

### Definitions

The CI-AKI was defined as ≥50% relative or ≥ 0.3 mg/dL absolute increase of SCr at 48 h after procedure [14]. Chronic kidney disease was diagnosed if eGFR was < 50 ml/min/1.73 m^2^ or in case of documented proteinuria > 500 mg/l. Hypertension was defined if mean blood pressure from two measurements was > 140/90 mmHg on two separate visits or antihypertensive agents regiment. The severity of CAD was reflected by SYNTAX score, which was calculated using the official online calculator by an experienced invasive cardiologist.

### Statistical analysis

Statistical calculations were processed using MedCalc v.14.8.1 software package (MedCalc, Ostend, Belgium). Continues variables were expressed as mean and standard deviation (SD) or median and 25–75 percentile boundaries, depending on the distribution, which was defined using Shapiro-Wilk’s test. The difference between MACCE and non-MACCE cohort was established by means of Student’s t test in case of normal distribution or Mann–Whitney U in the instance of non-normally distributed parameters. All the variables with *p* < 0.1 in univariate model were incorporated into the Cox proportional hazards model. Hazard ratio (HR) with 95% confidence intervals (95% CI) were calculated. The diagnostic power of different intra-renal flow parameters was established using the receiver operating characteristics (ROC) curve analysis. Cut-off values of different flow parameters were defined on the basis of Youden’s J statistic. Kaplan-Meier survival curves and log-rank tests were calculated. A *p*-value of less than 0.05 was deemed statistically significant across the analyses.

## Results

### General characteristics

The demographic and clinical characteristics of the population was highlighted in Table 1. The study population comprised 111 patients referred to elective or urgent coronary angiography with the diagnosis of stable angina (42.3%), or non-ST-elevation acute coronary syndrome (57.7%). The population was characterized by the predominance of males (68.5%). The population represented a typical cohort of CAD patients with median age of 65 (59; 71) years and pronounced cardiovascular risk factors, such as high prevalence of cigarette smoking (59.5%), hypertension (96.4%), diabetes (37.8%). Of note, history of past myocardial infarction in anamnesis was present in 45.9% of patients.

**Table 1:** Demographic and clinical characteristics of study population, stratified depending on the presence of MACCE during 24-month follow-up

Variable	Whole population ***n*** = 111	Non-MACCE ***n*** = 97	MACCE ***n*** = 14	***p***
Men	76 (68.5%)	65 (67.0%)	11 (78.6%)	0.29 ^a^
Age [years]	65 (59; 71)	65 (60; 71)	63.5 (57; 69)	0.97 ^b^
BMI [kg/m^2^]	29.1 ± 4.5	29.3 ± 4.6	27.8 ± 3.0	0.26 ^c+^
Waist circumference [cm]	90.6 ± 11.5	91.0 ± 11.9	87.7 ± 8.7	0.32 ^c^
Cigarette smoking	66 (59.5%)	57 (58.8%)	9 (64.3%)	0.47 ^a^
Hypertension	107 (96.4%)	93 (95.9%)	14 (100.0%)	0.58 ^a^
Diabetes type 2	42 (37.8%)	35 (36.1%)	7 (50.0%)	0.24 ^a^
Atrial fibrillation	24 (21.6%)	23 (23.7%)	1 (7.1%)	0.14 ^a^
History of MI	51 (45.9%)	44 (45.4%)	7 (50.0%)	0.48 ^a^
History of ischemic stroke/TIA	8 (7.2%)	8 (8.3%)	0 (0.0%)	0.26 ^a^
COPD	9 (8.1%)	9 (9.3%)	0 (0.0%)	0.23 ^a^
CI-AKI by AKIN criteria	9 (8.1%)	6 (6.2%)	3 (21.4%)	0.09 ^a^
NSTE-ACS	64 (57.7%)	54 (55.7%)	10 (71.4%)	0.27 ^a^
NSTEMI	32 (28.8%)	27 (27.8%)	5 (35.7%)	0.37 ^a^
Unstable angina	32 (28.8%)	27 (27.8%)	5 (35.7%)	0.37 ^a^
Left main CAD	11 (9.9%)	5 (5.2%)	6 (42.9%)	< 0.001 ^a^
SYNTAX score [pts]	13 (4; 25)	12 (3; 24)	22.5 (11; 36)	0.02 ^b^
PCI ad hoc	49 (44.1%)	42 (43.3%)	7 (50.0%)	0.64 ^a^
CABG referral	17 (15.3%)	13 (13.4%)	4 (28.6%)	0.14 ^a^
Hemoglobin [g/dL]	13.9 ± 1.24	14.0 ± 1.22	13.3 ± 1.23	0.07 ^c^
White blood cell count [×1000/mm^3^]	7.07 ± 1.73	7.11 ± 1.73	6.74 ± 1.78	0.46 ^c^
Platelet count [×1000/mm^3^]	201 (177; 254)	207.2 ± 0.02	242.4 ± 0.02	0.01 ^c^
Peak hsTnT [ng/mL]	0.013 (0.008; 0.034)	0.013 (0.008; 0.032)	0.023 (0.012; 0.052)	0.46 ^b^
Serum creatinine [mg/dL]	0.92 (0.79; 1.13)	0.92 (0.79; 1.11)	1.03 (0.83; 1.25)	0.23 ^b^
eGFR [ml/min/1.73 m^2^]	80.3 ± 20.8	80.5 ± 20.6	79.2 ± 23.4	0.83 ^c^
IMT [mm]	0.10 ± 0.03	0.09 ± 0.03	0.10 ± 0.02	0.17 ^c^
LVEF [%]	53.3 ± 7.3	53.2 ± 7.2	53.3 ± 7.7	0.99 ^c^
LVEF< 50%	30 (27.0%)	23 (23.7%)	7 (50.0%)	0.045 ^a^
E/e’	8.6 (6.7; 12.0)	8.6 (6.7; 12.1)	6.8 (6.4; 10.0)	0.06 ^b^
Mitral valve insufficiency	72 (64.9%)	66 (68.0%)	6 (42.9%)	0.07 ^a^
Systolic BP [mmHg]	130 (120; 140)	130 (120; 140)	140 (120; 150)	0.33 ^b^
Diastolic BP [mmHg]	80 (70; 90)	80 (70; 90)	80 (70;100)	0.15 ^b^
Pulse BP [mmHg]	40 (30; 50)	40 (30; 50)	40 (30; 55)	0.79 ^a^
Vmax – abdominal aorta [m/s]	0.60 ± 0.13	0.59 ± 0.13	0.61 ± 0.17	0.73 ^c^
RRI pre.	0.63 ± 0.07	0.62 ± 0.06	0.68 ± 0.06	< 0.001 ^c^
RRI post.	0.68 ± 0.07	0.66 ± 0.06	0.72 ± 0.06	0.01 ^c^
∆RRI	0.05 ± 0.05	0.05 ± 0.05	0.04 ± 0.04	0.42 ^c^
RPI pre.	1.40 (1.25; 1.52)	1.39 ± 0.20	1.36 ± 0.25	0.63 ^c^
RPI post.	1.51 (1.36; 1.64)	1.48 ± 0.21	1.54 ± 0.24	0.36 ^c^
∆RPI	0.10 ± 0.19	0.09 ± 0.20	0.18 ± 0.13	0.11 ^c^
PSV pre. [m/s]	0.42 ± 0.09	0.43 ± 0.09	0.40 ± 0.11	0.33 ^c^
PSV post. [m/s]	0.45 ± 0.08	0.45 ± 0.08	0.45 ± 0.10	0.83 ^c^
EDV pre. [m/s]	0.16 ± 0.05	0.16 ± 0.05	0.13 ± 0.05	0.01 ^c^
EDV post. [m/s]	0.15 ± 0.05	0.15 ± 0.05	0.12 ± 0.04	0.045 ^c^
AcT pre. [ms]	61 (51; 69.5)	57 (50.5; 68.5)	69.75 (58.5; 77.5)	0.06 ^b^
AcT post. [ms]	82 (68.5; 98.0)	77.5 (65.5; 98.0)	94.0 (85.5; 107.0)	0.11 ^b^
∆ AcT [ms]	18.5 (9.5; 32.5)	18.5 (9.5; 31.0)	19.0 (17.5; 38.0)	0.66 ^b^
AI pre. [m/s^2^]	4.1 (3.6; 4.6)	4.1 (3.7; 4.6)	3.7 (3.4; 4.2)	0.42 ^b^
AI post. [m/s^2^]	3.6 (3.1; 4.1)	3.5 (3.2; 4.0)	3.6 (3.0; 4.3)	0.90 ^b^
∆ AI [m/s^2^]	- 0.5 (0.9; 0.1)	−0.5 (−0.9; −0.1)	−0.5 (−0.9; −0.1)	0.36 ^b^

^a^Fisher’s exact test

^b^Mann-Whitney’s U test

^c^Student’s t test

*AKIN* Acute Kidney Injury Network criteria, *BMI* Body mass index, *COPD* Chronic obstructive pulmonary disease, *LVEF* Left ventricular ejection fraction, *MVI* Mitral valve insufficiency, *LM* Left main, *RRI* Renal resistive index, *RPI* Renal pulsatility index, *AI* Acceleration index, *AcT* Acceleration time, *PSV* Peak systolic velocity, *EDV* End-diastolic velocity, *NSTE-ACS* Non-ST-segment elevation acute coronary syndrome, *NSTEMI* Non-ST-elevation myocardial infarction, *CI-AKI* Contrast-induced acute kidney injury, *PCI* Percutaneous coronary intervention, *pre.* Pre-procedural, *post.* Post-procedural, *LVEF* Left ventricular ejection fraction, *hs-TnT* High sensitivity troponin T, *eGFR* Estimated glomerular filtration rate

### Major adverse cerebral and cardiovascular events

The total number of 14 MACCE events occurred in 14 patients (12.6%). Three patients experienced acute myocardial infarction; eight patients underwent urgent percutaneous myocardial revascularization due to unstable angina and ischemic stroke occurred in three patients.

The comparison of MACCE and non-MACE cohorts was presented in Table 1. Patients who exhibited MACCE more often had left main CAD (*p* < 0.001), higher SYNTAX score (*p* = 0.02) and platelet count (*p* = 0.01) and lower prevalence of depressed LVEF (*p* = 0.02). Both groups did not differ in terms of rate of myocardial infarction or ad hoc PCI.

Most importantly, MACCE cohort had higher pre- (0.68 ± 0.06 vs. 0.62 ± 0.06, *p* < 0.001) and post-procedural RRI (0.72 ± 0.06 vs. 0.66 ± 0.06, *p* = 0.01) and lower intra-renal pre- (0.13 ± 0.05 vs. 0.16 ± 0.05 m/s, *p* = 0.01) and post-procedural EDV (0.12 ± 0.04 vs. 0.15 ± 0.05 m/s, *p* = 0.045). Other intra-renal Doppler flow parameters did not differ between study groups (Table 1).

### ROC analysis and Kaplan-Meier survival curves

**Fig. 1:**
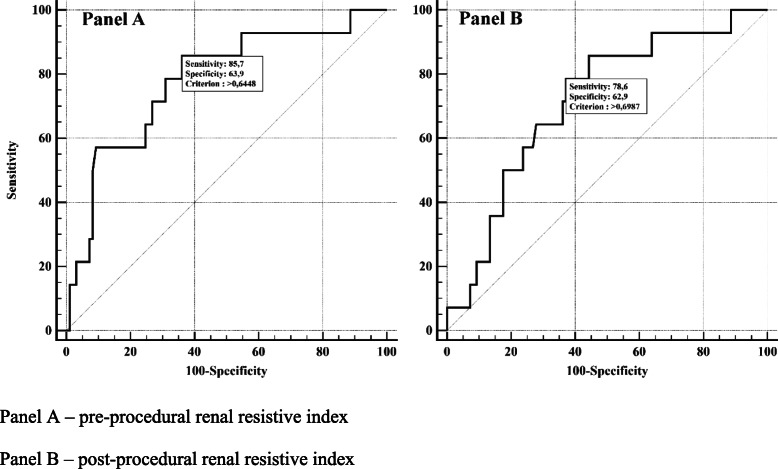
Receiver operating characteristics curve showing pre- and post-procedural renal resistive index as a predictor of major adverse cardiovascular events. **a** – pre-procedural renal resistive index. **b** – post-procedural renal resistive index

**Fig. 2:**
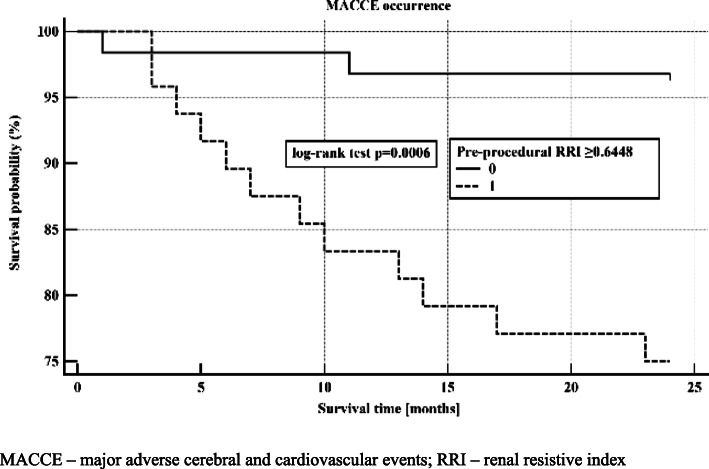
Kaplan-Meier survival curve of MACCE occurrence during 24-month observation depending on the level of pre-procedural RRI: ≥ or < the j-statistic threshold of 0.6448. MACCE – major adverse cerebral and cardiovascular events; RRI – renal resistive index

The ROC curve analysis was showed in Table 2. The analysis revealed that both pre- (AUC 0.780; *p* < 0.001; cut-off > 0.645; Fig. 1) and post-procedural (AUC 0.715; *p* = 0.003; cut-off > 0.699; Fig. 1) RRI had a good diagnostic power in prediction of MACCE. These threshold values of RRI were subsequently applied for Kaplan-Meier survival curves. Patients characterized by pre-procedural RRI > 0.645 had significantly higher risk of MACCE as reflected by Kaplan-Meier survival curve (log-rank *p* < 0.001; Fig. 2), as well as patients with post-procedural RRI > 0.699 (log-rank *p* = 0.004). Patients with pre-procedural intra-renal EDV < 0.13 m/s had also more favorable outcome (log-rank *p* = 0.006).

**Table 2:** Receiver operating characteristics curve analysis of different laboratory predictors of 12-month MACCE occurrence

Variable		Criterion	Sensitivity	Specificity	AUC	***p***
RRI	Baseline	> 0.645	85.7	63.9	0.780	< 0.001
	Post-proc. at 1 h	> 0.699	78.6	62.9	0.715	0.003
	∆	≤0.049	78.6	46.4	0.570	0.36
RPI	Baseline	≤1.185	35.7	84.5	0.500	0.99
	Post-proc. at 1 h	> 1.595	50.0	73.2	0.584	0.38
	∆	> 0.2	50.0	77.3	0.635	0.09
AI	Baseline	≤3.80	57.1	73.2	0.616	0.18
	Post-proc. at 1 h	≤3.13	42.9	80.4	0.522	0.80
	∆	> 0.74	14.3	100.0	0.523	0.79
AT	Baseline	> 71.5	50.0	83.5	0.663	0.06
	Post-proc. at 1 h	> 82	85.7	55.7	0.684	0.004
	∆	> 10	100.0	29.9	0.593	0.17
PSV	Baseline	≤0.27	21.4	95.9	0.542	0.64
	Post-proc.at 1 h	≤0.46	64.3	51.6	0.520	0.83
	∆	> 0.06	28.6	86.6	0.559	0.49
EDV	Baseline	≤0.13	64.3	72.2	0.706	0.01
	Post-proc.at 1 h	≤0.13	71.4	62.9	0.679	0.02
	∆	> − 0.02	42.9	41.2	0.503	0.98

*AI* Augmentation index, *AUC* Area under curve, *CAD* Coronary artery disease, *CI-AKI* Contrast-induced acute kidney injury, *HR* Hazard ratio, *CI* Confidence interval, *RPI* Renal pulsatility index, *RRI* Renal resistive index, *PSV* Peak systolic velocity, *EDV* End-diastolic velocity, *AT* Acceleration time

Also, the presence of left main CAD (log-rank *p* < 0.0001), but neither the diagnosis of NSTEMI (*p* = 0.52), nor PCI at index hospitalization (*p* = 0.67) stratified the population in terms of survival free from MACCE.

### Cox proportional hazards model

The results of univariate and multivariate Cox regression analysis are presented in Table 3. After inclusion of all statistically significant (or borderline; *p* < 0.1) variables, the final model yielded pre-procedural RRI (OR 1.11 per 0.01, 95%CI: 1.02–1.20, *p* = 0.02) and left main CAD (OR 5.75, 95%CI: 1.88–17.62, *p* = 0.002) as significant predictors of MACCE during 24-month follow-up.

**Table 3:** Univariate and multivariate Cox proportional hazards model of different predictors of MACCE onset during 24-monh follow-up

Variable	Univariate analysis			Stepwise multivariate Cox regression Overall model fit: ***p*** = 0.0001 Chi-square = 18.668		
	HR	95% CI	***p***	HR	95% CI	***p***
Male sex	1.77	0.50–6.30	0.38	–	–	–
Age [per 1 years]^a^	0.99	0.94–1.06	0.96	–	–	–
Arterial hypertension	21.31	< 0.001 – > 1000	0.62	–	–	–
Diabetes mellitus	1.68	0.59–4.78	0.33	–	–	–
Smoking	1.28	0.43–3.79	0.66	–	–	–
CI-AKI onset	3.56	1.00–12.73	0.05	–	–	–
Left main CAD	9.00	3.11–26.03	< 0.001	5.75	1.88–17.62	0.002
Syntax score [per 1 point]^a^	1.04	1.01–1.08	0.02	–	–	–
Platelet count [per 1/mm^3^]^a^	1.01	1.00–1.02	0.02	–	–	–
RRI pre. [per 0.01]^a^	1.14	1.05–1.22	0.001	1.11	1.02–1.20	0.02
RRI post. [per 0.01]^a^	1.11	1.02–1.21	0.02	–	–	–
EDV pre. [per 0.01 m/s]^a^	0.86	0.76–0.96	0.01		–	–
EDV post. [per 0.01 m/s]^a^	0.87	0.75–0.99	0.04	–	–	–
AT pre. [per 1 ms]^a^	1.02	1.00–1.04	0.06	–	–	–

^a^unit odds ratio

*CAD* Coronary artery disease, *CI-AKI* Contrast-induced acute kidney injury, *HR* Hazard ratio, *CI* Confidence interval, *RRI* Renal resistive index, *EDV* End-diastolic velocity, *AT* Acceleration time

## Discussion

The present study delivered evidence for significantly higher values of both pre- and post-procedural RRI in patients with MACCE than in patients with uneventful 24-month follow-up after coronary angiography (Table 1). The difference in RRI was predominantly triggered by markedly lower pre- and post-procedural EDV (Table 1). Cox proportional hazards model corroborated that pre-procedural RRI and presence of LM disease were independent predictors of MACCE at 24-month follow-up (Table 3). Patients with pre-procedural RRI above the threshold of 0.645 had significantly worse MACCE-free survival than patients with low levels of RRI according to Kaplan-Meier survival curve (Fig. 2).

These findings for the first time shed light on the prognostic aspect of Doppler-derived RRI, mediated by EDV as its crucial determinant, in patients with atherosclerotic cardiovascular disease, since it accurately identified patients at risk of future adverse cardiovascular events. In the study by Pearce and coworkers performed on a large cohort of 870 elderly patients (mean age of 77 ± 5 years) participating in Cardiovascular Health Study [17], end-diastolic velocity was inversely associated with the onset of composite endpoint of fatal or nonfatal myocardial infarction, hospitalization for angina, need for PCI or CABG, fatal or nonfatal stroke or transient ischemic attack (HR: 0.73, 95%CI: 0.62–0.87, *p* < 0.001). The RRI itself expressed as a qualitative variable (RRI ≥ or < 0.8) failed to predict onset of MACCE (HR 1.10, 95%CI: 0.81–1.50, *p* = 0.5). Still, RRI is dependent on EDV value and presumably RRI should be treated as a continuous variable, as opposed to using an arbitrary high threshold of 0.8. In our study, pre-procedural RRI values as low as 0.645 stratified the population in terms of long-term outcome (Fig. 2).

The report by Doi et al. delivered proof that RRI is predictive of adverse cardiovascular events in a broad group of patients with arterial hypertension [18]. This study covered 426 patients without cardiovascular disorders in anamnesis and found that RRI was associated with MACCE occurrence (HR 1.81 for 1 SD increase, 95%CI: 1.45–2.27, *p* < 0.01) [18]. The prognosis was worse among patients with RRI ≥ 0.65 for males and 0.68 for females (log rank *p* < 0.01) [18]. Contrary to the cited results, our study enrolled patients with symptomatic CAD, however, the vast majority of patients (96.4%) also had hypertension. Unlike our research, which used CKD as an exclusion criterion, the study by Doi enrolled 133 patients with eGFR < 60 ml/min/1.73 m^2^, precluding direct comparison of its results [18]. It is vital to note that RRI exhibited higher predictive power among patients with CKD as opposed to patients with preserved renal function (HR 2.11 for 1 SD increase, 95%CI: 1.44–3.16, *p* < 0.01).

The predicate role of RRI in patients with impaired renal function and arterial hypertension was confirmed in the study Toledo et al. Based on a cohort of 1962 patients with CKD (eGFR 15–59 ml/min), the authors provided evidence that RRI > 0.70 was associated with higher long-term mortality (HR 1.29, 95%CI: 1.02–1.65, *p* < 0.05), especially in younger group and in patients with stage 3 CKD [19]. This study was the only trial, which unequivocally linked RRI to mortality [19], not only to composite endpoint.

The results of the current research are novel as the study exclusively covered patients with preserved renal function (eGFR ≥50 ml/min/1.73 m^2^), hence it highlights the clinical benefits of RRI assessment regardless of baseline kidney condition, which could alter the results of RRI.

The mechanism underlying the predictive role is presumably related to its association with structural vascular remodeling (stiffness) and sympathetic tone (renal artery constriction). The current study design is unique as it compensated for the possible variations of intra-renal flow parameters by excluding patients with extreme values of heart rate, pulse blood pressure or valvular heart disease. All these parameters remain significant determinants of RRI and also long-term outcome. Of note, pulse blood pressure represents a strong predictor of cardiovascular outcome and RRI is highly dependent on its value [10]. In our study, pulse blood pressure was comparable between both groups, yet RRI was not and thus other mechanisms drove higher RRI values in patients at risk of future adverse cardiovascular events. Presumably higher sympathetic tone within renal arcuate and interlobular arteries might contribute to lower EDV and higher RRI values in high risk patients. It is meaningful that pre-procedural values, without the deleterious impact of contrast media, had the highest predictive value towards long-term outcome.

These results correspond with former studies in the field, which showed association of higher level of cardiovascular risk factors among patients with higher RRI values [9, 10, 21]. Prejbisz et al. revealed that patients with truly resistant hypertension had higher RRI values than patients with well-controlled arterial hypertension (0.62 ± 0.05 vs. 0.60 ± 0.05, *p* < 0.05) [21]. RRI exhibited correlation with ambulatory blood pressure, pulse blood pressure, fasting glucose concentration and E/e’ ratio [21]. The metabolic aspect of RRI was documented on the basis of patients with type 2 diabetes, in whom dynamic RRI (change of RRI following administration of sublingual nitrate administration) predicted onset of microalbuminuria [22].

Last but not least, Geraci et al. delineated that RRI values were associated with the extent of carotid atherosclerosis [23]. In their study RRI correlated with carotid IMT assessed in patients with hypertension, both with (*r* = 0.42) and without CKD (*r* = 0.39) [23]. More recently, the same group of authors provided evidence that Doppler-derived intra-renal flow parameters were associated with the severity of coronary atherosclerosis assessed in coronary angiography, but only among patients with less pronounced atherosclerosis [24]. Of note, this association was valid for RPI, but not RRI, which requires further explanation [24].

In the former study conducted in our institution, RRI was documented to be an independent predictor of CI-AKI [14]. Although CI-AKI rate was higher in MACCE cohort in the present analysis, it did not reach statistical significance and impaired prognosis cannot be solely attributed to this covariate [14]. Conversely, the presence of LM disease was a powerful predictor of event-free survival (HR 5.75, 95%CI: 1.88–17.62, *p* = 0.002), which corresponds with general risk stratification demonstrated in large clinical trials.

### Study limitations

The main disadvantage of the study is limitation of RRI itself, as it depends on numerous variables. Still, subjects with extreme values of possible determinants of RRI [25], such as elderly age, severe tachy- and bradycardia, high pulse pressure were excluded from the study, which prevented hypothetical impact of these variables on study results. Also, the measurement of PSV and EDV and its derivatives is limited by rather high inter- and intra-observer variability. We attempted at limiting this shortcoming by conducting repeated measurement in both kidneys and using an arithmetic mean of all measurements.

## Conclusions

Increased pre-procedural renal resistive index, together with left main CAD, are linked to 24-month prognosis in patients with CAD referred for coronary catheterization. This easily accessible diagnostic tool could improve the stratification of cardiovascular risk in CAD patients referred for invasive procedures.

## Data Availability

Upon notice, the authors are willing to share their raw data with all the interested parties.

## Abbreviations

AKIN: Acute kidney injury network criteria
AcT: Acceleration time
AI: Acceleration index
AUC: Area under curve
BMI: Body mass index
CA: Coronary angiography
CABG: Coronary artery bypass grafting
CAD: Coronary artery disease
CI: Confidence interval
CI-AKI: Contrast-induced acute kidney injury
CKD: Chronic kidney disease
COPD: Chronic obstructive pulmonary disease
eGFR: Estimated glomerular filtration rate
HR: Hazard ratio
LM: Left main
LVEF: Left ventricular ejection fraction
MACCE: Major adverse cardiac and cerebrovascular events
MVI: Mitral valve insufficiency
NSTE-ACS: Non-ST-segment elevation acute coronary syndrome
NSTEMI: Non-ST-elevation myocardial infarction
PCI: Percutaneous coronary intervention
ROC: Receive operating characteristics curve
RRI: Renal resistive index
RPI: Renal pulsatility index
PSV: Peak systolic velocity
EDV: End-diastolic velocity
pre: Pre-procedural
post: Post-procedural
hs-TnT: High sensitivity troponin T
SCr: Serum creatinine concentration
SD: Standard deviation
